# Infant Feeding Practices, Prevalence of Adolescent Motherhood and Malnutrition among Infants in Mangu Local Government Area, Plateau State, Nigeria

**DOI:** 10.5334/aogh.4229

**Published:** 2024-07-19

**Authors:** Aloysius N. Maduforo, Clementina E. Okoro, Justina N. Chikwendu, Chika Ndiokwelu, Gift Asogwa, Miracle C. Aloysius-Maduforo, Chinyere C. Okwara, Josephine Nwanneoma Okorie

**Affiliations:** 1 Department of Nutrition and Dietetics, University of Nigeria, Nsukka, Nigeria; 2 Department of Educational Research (Leadership), Werklund School of Education, University of Calgary, Alberta, Canada; 3 Nutrition Section, Federal Capital Territory Primary Health Care Board, Abuja, Nigeria; 4 Department of Human Nutrition and Dietetics, University of Calabar, Nigeria; 5 Department of Educational Research (Learning Sciences), Werklund School of Education, University of Calgary, Alberta, Canada; 6 Department of Nutrition and Dietetics, University of Nigeria Teaching Hospital Ituku-Ozalla, Enugu State, Nigeria; 7 Niger State Polytechnic, Zungeru, Niger State, Nigeria; 8 School of Community Health and Policy, Morgan State University, Maryland, United States

**Keywords:** Adolescent motherhood, malnutrition, infants, IYCF

## Abstract

*Background:* Adolescent motherhood and malnutrition among children are significant challenges in Africa, but there is limited data on the impact of adolescent motherhood on their children’s health and nutrition. This study assessed infant feeding practices, prevalence of adolescent motherhood, and malnutrition among infants in Mangu local government area (LGA).

*Methodology:* A cross-sectional survey using multistage sampling was conducted. Validated questionnaires were used to collect socio-demographic data, and appropriate tools were used for anthropometric measurements. Data were compared with established standards. Descriptive statistical tools, chi square, Pearson correlation, and independent sample *t*-test were used for data analysis, with significance set at *p* < 0.05.

*Results:* A total of 200 mothers completed the study. The majority of the infants (78.5%) were less than 6 months old, and 21.5% were 6–12 months old. Breastfeeding initiation within 1 hour was reported by 39% of mothers, while 38% practiced prelacteal feeding. Only 28.5% practiced exclusive breastfeeding, and all mothers breastfed their babies. The prevalence of adolescent motherhood was 37.5%. The prevalence of stunting, wasting, and underweight among infants were 29.5%, 12%, and 8.5%, respectively. Children of adolescent mothers had higher rates of severe stunting compared to children of mothers above 19 years of age. There were significant differences (*p* = 0.017 and *p* = 0.029) in stunting rates and weight-for-age indices between children of adolescent mothers and mothers above 19 years of age.

*Conclusion:* Adolescent motherhood contributes to chronic malnutrition in children, and there is a high prevalence of malnutrition among infants in Mangu LGA, Plateau State.

## Introduction

Child marriage, teenage pregnancy, and adolescent pregnancy have been traditional and religious practices in many countries, including Nigeria. Adolescent mothers are young women between the ages of 10 to 19 who are nursing their own biological child [1]. Proper nutrition during infancy and early childhood is crucial for the optimal development of a child’s full potential [2]. The period from birth to two years of age is recognized as a “critical window” for promoting optimal growth, health, and behavioral development. Malnutrition during early life can increase the risk of infections, morbidity, and mortality, as well as decrease mental and cognitive development [3, 4]. The effects of child malnutrition can have long-lasting impacts that extend beyond childhood, such as being linked to communicable and non-communicable diseases in adults worldwide [5], and low work productivity in later life [3]. Thus, ensuring adequate nutrition during infancy and early childhood is essential for the health, growth, and development of infants to their full potential [6].

Undernutrition, as a form of malnutrition, is prevalent in all societies worldwide, with mothers and infants being at the greatest risk [7]. Undernutrition is the most common form of malnutrition in sub-Saharan Africa (SSA) and is highly prevalent in many developing countries [7–9]. The 2018 Nigerian National Nutrition and Health Survey reported that the national prevalence of global acute malnutrition (GAM), defined as weight-for-height/length (WHZ < −2 or edema), for children aged 6–59 months was 7%; moderate acute malnutrition (MAM) (WHZ ≥ −3 and ≤ −2) was 5.5%; and severe acute malnutrition (SAM), defined as WHZ <−3 and/or edema, was 1.5% [10].

Mangu LGA is located in Plateau State, north-central Nigeria, and is the largest settlement in terms of population, serving as the administrative headquarters of the local government. A study conducted by Hyeladi and Alfred in 2014 [11] reported that the poverty rate in Mangu LGA was 92% based on dollar rating, indicating a high prevalence of poverty. Economic status is a critical determinant of the nutritional status of households.

Adolescent females are still growing and are considered a vulnerable group for malnutrition. Their nutritional needs for growth increase during adolescence, and being pregnant and breastfeeding as adolescents further increases their vulnerability, potentially exposing their children to malnutrition due to various factors. Although some studies have discussed the issues associated with adolescent pregnancy, there is limited literature on the effect of adolescent motherhood on the nutritional status of their children [12–15]. It is crucial to routinely assess the prevalence of adolescent motherhood and pregnancy in communities to evaluate intervention programs by government agencies and civil society organizations in Nigeria. Therefore, this study assessed infant feeding practices, the prevalence of adolescent motherhood, and malnutrition among infants in Mangu LGA, Plateau State.

## Methodology

### Study area

The study was conducted in Mangu LGA in Plateau State, Nigeria, specifically in the town of Mangu at 9°31′00″N 9°06′00″E. Mangu LGA has an area of 1,653 km² and a population of 294,931 as per the 2006 census. The main languages spoken in Mangu are Mwaghavul and Pyem. There are 81 health centers and 10 district towns in Mangu LGA, namely Mangu district with 42 villages, Ampang west district with 24 villages, Mangun district with 37 villages, Kombun district with 36 villages, Panyam district with 30 villages, Kerang district with 15 villages, Langai district with 30 villages, Gindiri district with 40 villages, Pushit district with 20 villages, and Chakfem district with 12 major villages. The main source of income for the inhabitants is agriculture.

### Study design

The study followed a cross-sectional design and focused on infants in Mangu LGA.

### Study population

The sample population for the study consisted of women of childbearing age and their infants who visited the health centers in the study area. Mangu LGA has a total of 86,980 registered women of childbearing age, and this population size, along with their infants, was used for the study.

### Sampling techniques

A multistage sampling technique was employed to select the respondents. In the first stage, three districts were selected through balloting without replacement out of the ten districts in Mangu LGA. In the second stage, ten health centers were visited from each of the three districts, resulting in a total of thirty health centers. Convenience sampling was then used to select respondents at each health center.

### Informed consent

Participants were informed about the study and provided with a clear understanding of the procedures and potential risks involved. Participation was voluntary, and individuals had the right to choose whether or not to take part in the study. Only those who provided consent were included in the interviews and measurements.

## Data Collection

**Techniques and Tools:** Infant length was measured using a wooden infantometer for recumbent length, and weight was measured using a Seca digital weighing scale. This was done by nurses and the fourth-year student of nutrition and dietetics. All measurements followed the standard protocols and cut-offs for classifications set by the World Health Organization. Information on the socio-demographic characteristics of the infants was collected using a structured and validated questionnaire. Anthropometric indices were compared and classified according to the World Health Organization Z-score standard.

### Statistical analysis

Data were analyzed using descriptive statistical tools such as frequency tables, cross-tabulation, and bar charts. The chi-square test was used to assess relationships between categorical variables, while Fisher’s Exact Test was employed for small sample sizes to ensure accurate *p*-values. The significance level was set at *p* < 0.05.

## Results

The sociodemographic characteristics of the respondents are summarized in Table 1. A total of 200 respondents participated in the study, including 145 female children and 55 male children. The table indicates that the majority of infants (78.5%) were less than 6 months old, while 21.5% were aged 6–12 months. In terms of mothers’ occupations, 50.5% were traders. Additionally, 79% of the respondents identified as Christians, while 21% identified as Muslims.

**Table 1:** Socio-demographic characteristics of mothers of infants in Mangu LGA (*N* = 200, Females = 145; Males = 55).

VARIABLES	FEMALE F (%)	MALE F (%)	TOTAL F (%)	*P*-VALUE
Age Group				0.651
<6 months	115 (79.3)	42 (76.4)	157 (78.5)	
6–12 months	30 (20.7)	13 (23.6)	43 (21.5)	
Mother’s Occupation				0.884
Student	28 (19.3)	8 (14.5)	36 (18.0)	
Civil servant	28 (19.3)	11 (20.0)	39 (19.5)	
Trader	71 (49.0)	30 (54.5)	101 (50.5)	
Farmer	3 (2.1)	1 (1.9)	4 (2.0)	
Unemployed	15 (10.3)	5 (9.1)	20 (10.0)	
Mother’s Educational Level				0.093
No formal education	1 (0.7)	2 (3.6)	3 (1.5)	
Primary school uncompleted	1 (0.7)	1 (1,8)	2 (1)	
Primary school completed	7 (4.8)	3 (5.5)	10 (5)	
Secondary school uncompleted	21 (14.5)	9 (16.4)	30 (15)	
Secondary school completed	73 (50.3)	20 (36.4)	93 (46.5)	
Tertiary	42 (29.0)	20 (36.4)	62 (31.0)	
Parent’s Marital Status				0.522
Married	127 (87.7)	49 (89.1)	176 (88.0)	
Single	6 (4.1)	2 (3.6)	8 (4.0)	
Widow/widower	5 (3.4)	3 (5.5)	8 (4.0)	
Divorced	1 (0.7)	1 (1.8)	2 (1.0)	
Separated	6 (4.1)	0 (0.0)	6 (3.0)	
Religion	**0.046**	**0.831**		0.831
Christianity	114 (78.6)	44 (80.0)	158 (79.0)	
Islam	31 (21.4)	11 (20.0)	42 (21.0)	

Across various demographic and socioeconomic variables, including age group, mother’s occupation, mother’s educational level, parent’s marital status, and religion, there are no statistically significant differences between female and male infants in the Mangu LGA. The chi-square tests yielded the following *p*-values: age group (χ² = 0.205, *p* = 0.651), mother’s occupation (χ² = 1.164, *p* = 0.884), mother’s educational level (χ² = 12.242, *p* = 0.093), parent’s marital status (χ² = 3.220, *p* = 0.522), and religion (χ² = 0.046, *p* = 0.831). All *p*-values are greater than 0.05, indicating no statistically significant associations between these variables and the sex of the infants.

Table 2 provides an overview of the infant feeding practices among mothers in Mangu LGA, Plateau State. The results reveal that 39% of mothers initiated breastfeeding within 1 hour of childbirth, which is in line with national and global recommendations. However, 38% practiced prelacteal feeding, which means they fed their infants substances other than breastmilk before initiating breastfeeding. The exclusive breastfeeding rate was 28.5%, indicating that all mothers breastfed their babies, but only 28.5% introduced complementary foods at the appropriate age.

**Table 2:** Infant feeding practices of Mothers in Mangu LGA, Plateau State (*N* = 200, Females = 145; Males = 55).

VARIABLE	FREQUENCY (%)
Breastfeeding Initiation	
Within 1 hour	78 (39.0%)
Within 24 hours and above	122 (61.0%)
Practiced Prelacteal Feeding	
Yes	76 (38.0%)
No	124 (62.0%)
First Food/Fluid Given to the Infants	
Breastmilk only	124 (62.0%)
Warm water	62 (31.0%)
Glucose water	14 (7.0%)
Ever Breastfed	
Yes	200 (100.0%)
No	0 (0.0%)
Practiced Exclusive Breastfeeding	
Yes	57 (28.5%)
No	143 (71.5%)
Age of Complementary Food Introduction	
0–3 months	35 (17.5%)
>3 months – <6 months	32 (16.0%)
At 6 months	57 (28.5%)
7–12 months	32 (16.9%)
>12 months	2 (1.0%)

Table 3 presents the prevalence of adolescent motherhood in Mangu LGA, Plateau State. The table reveals that 62.5% of infant mothers were above 19 years of age at the time of their first child’s birth, while 84.5% of infant mothers were 19 years old during the study. Early adolescent motherhood (ages 10–15 years) accounted for 4% of first child births, while late adolescent motherhood (ages 16–19 years) accounted for 33.5%. The overall prevalence of adolescent motherhood at the time of the mother’s first child in the study was 37.5%, which decreased to 15.5% at the time of the study.

**Table 3:** Prevalence of adolescent motherhood in Mangu LGA, Plateau State (*N* = 200, Females = 145; Males = 55).

VARIABLES	FEMALE F (%)	MALE F (%)	TOTAL F (%)	*P*-VALUE
Adolescent motherhood at first childbirth				0.653
10–15 years	7 (4.8)	1 (1.8)	8 (4.0)	
16–19 years	46 (31.8)	21 (38.2)	67 (33.5)	
>19 years	92 (63.4)	33 (60.0)	125 (62.5)	
Adolescent motherhood at present child				0.279
10–15 years	2 (1.4)	0 (0)	2 (1.0)	
16–19 years	18 (12.4)	11 (20.0)	29 (14.5)	
>19 years	125 (86.2)	44 (80.0)	122 (84.5)	

The chi-square tests for adolescent motherhood at first childbirth (χ² = 0.202, *p* = 0.653) and at present childbirth (χ² = 1.173, *p* = 0.279) indicate no statistically significant differences between female and male infants. All *p*-values are greater than 0.05, suggesting no significant associations between the age of mothers at childbirth and the sex of the infants.

Table 4 outlines the prevalence of malnutrition among infants in Mangu LGA, showing that 29.5% were stunted, 12% were wasted, and 8.5% were underweight. Statistical analysis reveals no significant differences between female and male infants in the prevalence of stunting, wasting, or underweight conditions.

**Table 4:** Prevalence of malnutrition among infants in Mangu LGA (*N* = 200, Females = 145; Males = 55).

VARIABLE	FEMALE F (%)	MALE F (%)	TOTAL F (%)	*P*-VALUE
**Length-for-Age**				0.112
Severe stunting	13 (9.0)	5 (9.1)	18 (9.0)	
Moderate stunting	35 (24.1)	6 (10.9)	41 (20.5)	
Normal	97 (66.9)	44 (80.0)	141 (70.5)	
**Weight-for-Length**				1.000
Severe wasting	5 (3.4)	2 (3.6)	7 (3.5)	
Moderate wasting	13 (9.0)	4 (7.3)	17 (8.5)	
Normal	127 (87.6)	49 (89.1)	176 (88.0)	
**Weight-for-Age**				0.573
Severe underweight	1 (0.7)	1 (1.8)	2 (1.0)	
Moderate underweight	12 (8.3)	3 (5.5)	15 (7.5)	
Normal	132 (91.0)	51 (92.7)	183 (91.5)	

Across the various categories of malnutrition, there are no statistically significant differences between female and male infants. The chi-square test for Length-for-Age (χ² = 4.376, *p* = 0.112) and Fisher’s Exact Tests for Weight-for-Length (*p* = 1.000) and Weight-for-Age (*p* = 0.573) all indicate that the differences in malnutrition prevalence between female and male infants are not statistically significant.

**Figure 1:**
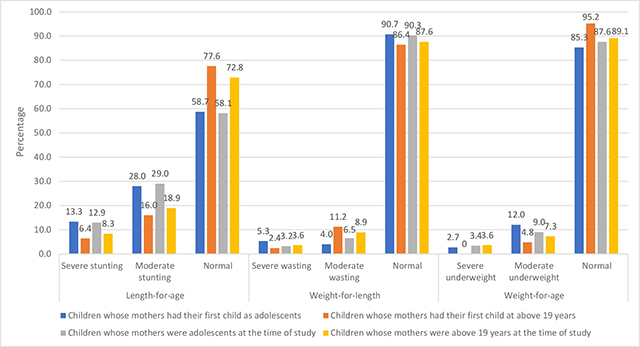
Prevalence of Malnutrition among Children of Adolescent Mothers (*N* = 200, Females = 145; Males = 55). The results of the correlation analysis indicated a significant positive correlation (*r* = 0.252; *p* = 0.000) between the weight of infants and the duration of breastfeeding.

Figure 1 and Table 5 provide a comparison of malnutrition levels among children born to adolescent mothers and non-adolescent mothers. The comparison also includes malnutrition prevalence among children whose mothers were adolescents at the time of their first child. The stunting rates of the children indicate that children of adolescent mothers had higher rates of severe stunting compared to children of mothers above 19 years of age. During the study, 12.9% of children of adolescent mothers were severely stunted, while 29% were moderately stunted. In contrast, children of mothers above 19 years of age had lower rates of severe and moderate stunting, with 8.3% and 18.9%, respectively. The stunting rates of children born to adolescent mothers and mothers above 19 years of age at the time of their first childbirth showed a significant difference (*p* = 0.017).

**Table 5:** The statistical relationship between the nutrition indices of children of adolescent mothers and mothers above 19 years of age (*N* = 200, Females = 145; Males = 55).

NUTRITION INDICES	AGE OF MOTHER AT FIRST CHILDBIRTH*χ*^2^ (*P*-VALUE)	AGE OF MOTHER AT THE TIME OF STUDY*χ*^2^ (*P*-VALUE)
Length-for-age	8.180 (0.017*)	2.729 (0.256)
Weight-for-length	4.108 (0.128)	0.900 (0.211)
Weight-for-age	7.072 (0.029*)	5.932 (0.052)

The statistical relationship is significant (*) when p < 0.05.

### Discussion

Chronic undernutrition, including stunting and underweight, was significantly higher among children born to adolescent mothers in this study, as compared to mothers above 19 years of age. This indicates the importance of physical, emotional, and biological maturity of women before becoming mothers. Most adolescents are still in school, and early marriage and/or pregnancy can hinder their academic progress and fulfillment of life goals. Ensuring that adolescents complete their education can enhance their ability to care for themselves and their children by increasing their earning power.

The purpose of this study was to assess the prevalence of adolescent motherhood and malnutrition among infants in Mangu LGA, Plateau State, Nigeria. Previous research has shown that the educational status of parents is associated with the nutritional status of their children, as educated parents tend to provide better infant feeding and care practices [16–19]. In the present study, a high percentage of mothers (77.5%) and fathers (63.1%) had completed secondary and higher levels of education. This contrasts with a study conducted in Bangladesh, where only 15.47% of mothers and 20.09% of fathers had completed secondary or higher levels of education, and this adversely affected the nutritional status of their children [18]. The results of the present study were also higher than those reported by Muhammad et al. [20] in Kano State, Nigeria, where only 46.5%, 24.8%, and 4% of fathers had, respectively, attained primary, secondary, and tertiary education as their highest education level. The disparity in results could be attributed to environmental influences and cultural values.

The study findings suggest that inadequate diet and disease are closely linked to the general standard of living, as families with more resources are better able to cater to the nutritional needs of their children. In the present study, most parents were employed but earned less than N 20,000 monthly, which is less than 50 US$ and amounts to less than $2 a day for a household. This low monthly income puts most families at risk of food insecurity, as they lack economic access to sufficient, safe, and nutritious food that meets their dietary and food preferences. Poverty is a significant risk factor for malnutrition, as the income of the household determines their purchasing power to obtain food [21]. However, it was found that many community members engage in farming to supplement their paid employment by producing some of the food their families consume, which partly cushions the effect of low income levels among the respondents.

The findings of this study revealed that 39% of mothers appropriately initiated breastfeeding within 1 hour of childbirth, which is comparable to the national level of 42% [22]. In contrast, the 2018 Nigeria Demographic and Health Survey (NDHS) [22] reported that 49% of children received prelacteal feed, which is higher than the 38% reported in this study. The national exclusive breastfeeding rate was 29% [22], which is similar to the rate of 28.5% reported in this study. While the 2018 NDHS reported that 97% of children were ever breastfed, in this study, all mothers breastfed their babies. It is important to note that breastfeeding and breastmilk remain the most appropriate method and food for children under 6 months of age [2, 22–25]. The timing of introduction of complementary foods is crucial in determining the rate of optimal exclusive breastfeeding in any country [2, 23, 24]. Globally, the recommendation is to introduce age-appropriate complementary foods when the child is 6 months old. In Mangu, 28.5% of mothers introduced complementary foods at 6 months of age, which is similar to the 29% reported in the 2018 NDHS [26].

In the present study, the prevalence of stunting, wasting, and underweight were 29.5% (severe 9% and moderate 20.5%), 12% (severe 3.5% and moderate 8.5%), and 8.5% (severe 1% and moderate 7.5%), respectively. These results were significantly lower than the national rates, except for wasting, where severe stunting, wasting, and underweight were reported to be 45%, 8%, and 27%, respectively [26]. These prevalence rates were also lower than those reported by Neima et al. [3] in a study on malnutrition prevalence and associated factors among children in rural Ethiopia, where underweight, wasting, and stunting were reported to be 27%, 9.7%, and 41.2%, respectively. Similarly, discordant findings were observed in a study conducted among children aged 6–60 months in eastern peri-urban communities of Nigeria, where the prevalence of stunting was 12.3% (10.6% moderately stunted and 1.7% severely stunted), and underweight was 17.4% (14% moderately underweight and 3.4% severely underweight) [27]. Ibeanu et al. [27] also reported a higher prevalence of underweight among children aged 6–24 months, which could be attributed to the transition from exclusive breastfeeding to complementary feeding. This high prevalence of underweight may be due to inadequate quantity and quality of traditional complementary foods in Nigeria to meet the nutritional needs of this age group [27]. Other studies conducted in Kano State, Nigeria, showed that 60.3% of children under 5 years of age had one or more forms of undernutrition [28]. The disparity in prevalence rates may be attributed to differences in socioeconomic status, culture, feeding habits, environmental factors, and utilization of public services in the study communities [29, 30].

In terms of stunting, males showed a slightly higher prevalence compared to females, although this difference was not statistically significant. This finding is consistent with results reported by other authors [27, 31, 32]. However, there is inconsistency in the literature regarding the association between gender and prevalence of malnutrition, as some studies have reported a higher prevalence of malnutrition among females [33], while others found no significant association [34].

Maternal and child health is a significant concern, particularly among adolescent mothers [12]. Globally, adolescent mothers are at higher risk of premature birth and low birth weight, which are risk factors for undernutrition in children [1, 35]. In this study, 37.5% of mothers were adolescents, and the prevalence of adolescent motherhood was 15.5%. This prevalence was lower than the findings in Wogedi, Northeast Ethiopia, where adolescent pregnancy prevalence was 28.6% [36]. However, a study in Ganye LGA, Adamawa State, Nigeria, reported a higher prevalence of 51% for adolescent pregnancy [12]. Adolescent motherhood is discouraged due to its negative impacts on medical, psychological, nutritional, and social aspects of adolescents [1, 12]. The practice of adolescent and child marriage, which often leads to adolescent pregnancy in northern Nigeria, is influenced by cultural, attitudinal, and religious factors, particularly Islam [12, 35].

## Conclusion

The study reveals a high prevalence of adolescent motherhood and malnutrition among infants in Mangu LGA, Plateau State. There is a critical need for nutrition and health education at the primary and secondary schools, as well as community level, to raise awareness about the risks of adolescent motherhood and promote proper infant and young child feeding practices.

## References

[r1] WHO. Making Pregnancy Safer. 2011. http://www.who.int/making_pregnancy_safer/topi%0Acs/adolescent_pregnancy/en/index.htm.

[r2] WHO. Global Strategy for Infant and Young Child Feeding. UNICEF. 2003. 15806879

[r3] Neima E, Henok A, Lamessa D. Prevalence of malnutrition and associated factors among children in rural Ethiopia. Biomed Res Int. 2017;2017:6587853. 28596966 10.1155/2017/6587853 PMC5449753

[r4] World Health Organization. Essential Nutrition Actions: Improving Maternal, Newborn, Infant and Young Child Health and Nutrition. WHO, Geneva; 2013. https://www.who.int/publications/i/item/9789241505550. 25473713

[r5] Ashabam S, Rukundo GZ, Beinempaka F, Ntaro M, Lebnac J. Maternal depression and malnutrition in children in southwest Uganda: A case control study. BMC Public Health. 2015;15:1–6. 26712120 10.1186/s12889-015-2644-y PMC4693407

[r6] Giao H, Quynh H, Ngoc H, Quang T, Van Khanh T. Malnutrition among 6–59 month-old children at District 2 Hospital, Ho Chi Minh City, Vietnam: Prevalence and associated factors. Biomed Res Int. 2019;2019:1–8. 10.1155/2019/6921312 PMC6379874 30868070

[r7] Black P, Victoria C, Walker S. The Maternal and Child Nutrition Study Group. Maternal and child undernutrition and overweight in low and middle-income countries. Lancet. 2013;382(9890):427–451. doi:10.1016/s0140-6736(13)60937-x. 23746772

[r8] Triunfo S, Lanzone A. Impact of maternal undernutrition on obstetric outcomes. J Endocrinol Invest. 2014;38(1):1–18. doi:10.1007/s40618-014-0168-4. 25194427

[r9] UNICEF. Types of Undernutrition: Growth Failure. 2009. http://conflict.lshtm.ac.uk/page_115.htm.

[r10] National Bureau of Statistics. National Nutrition and Health Survey; Report on the Nutrition and Health Situation of Nigeria. Abuja; 2018. https://nigerianstat.gov.ng/pdfuploads/NNHS_2018_Final%20Report.pdf

[r11] Hyladi A, Alfred J. Assessment of family size and poverty levels in Mangu LGA, Plateau State. Int J Humanit Soc Sci. 2014;4(3):310–315.

[r12] Maduforo AN, Ojebode O. Prevalence of adolescent pregnancy in Ganye local government area, Adamawa State, Nigeria. JORIND. 2011;9(2):123–134. https://pdfs.semanticscholar.org/9b14/bdfd21b0f98f823b7ee62ec771c8cb54dc9f.pdf?_ga=2.35150945.1715690293.1585228486-106976840.1584128875.

[r13] Yassin SA, Sobhy SI, Ebrahim W. Factors affecting dietary dietary practice among adolescent pregnant women in Alexandria. J Egypt Public Health Assoc. 2004;79:179–196. 16918146

[r14] Gutierrez YM. Cultural factors affecting diet and pregnancy outcome of Mexican American adolescent. J Adolesc Health. 1999;25:943–953. 10.1016/s1054-139x(99)00016-6 10475499

[r15] Ismail MK, Umar IU, Garba DG, Muutassim I. Prevalence of childhood and adolescent overweight and obesity in Kano State, Nigeria. EC Paediatr. 2018;7(4):231–238.

[r16] Khattak UK Iqbal SP, Ghazanfar H. The role of parent’s literacy in malnutrition of children under the age of five years in a semi-urban community of Pakistan: A case-control study. Cureus. 2017;9(6):e1316. doi:10.7759/cureus.1316. 28690950 PMC5498125

[r17] Ansuya B, Nayak B, Anice G, Shashidhara Y, Suneel C, Vasudev G. Risk factors of malnutrition among preschool children in rural Karnataka: A case-control study. BMC Public Health. 2018;2018(18):283. 10.1186/s12889-018-5124-3 PMC5828120 29482540

[r18] Belal H, Hasinur R. Role of parental education in reduction of prevalence of childhood undernutrition in Bangladesh. Public Health Nutr. 2018;21(10):1845–1854. doi:10.1017/S1368980018000162. 29455704 PMC10260740

[r19] Pragati C, Mukta A. Malnutrition and associated factors among children below five years of age residing in slum area of Jaipur City, Rajasthan, India. Asian J Clin Nutr. 2019;11:1–8.

[r20] Muhammad A, Yunusa I, Bolori M, Ezeanyika I, Walla H, Gidado Z. Malnutrition among children under 5 does not correlate with higher socio economic status of parents in rural communities. Open Access Lib J. 2017;4:1–15.

[r21] FAO. *Baseline Survey Report Protecting and Improving Household Food Security and Nutrition in* HIV/AIDS *Affected Areas in Mania and Sofala Province*. 2006. https://www.fao.org/fileadmin/user_upload/eufao-fsi4dm/doc-training/baseline_june07.pdf.

[r22] National Population Commission [Nigeria], ICF. Nigeria Demographic Health Survey 2018. 2019. https://dhsprogram.com/publications/publication-fr359-dhs-final-reports.cfm.

[r23] WHO/UNICEF. Baby Friendly Hospital Initiative: Revised, Updated and Expanded for Integrated Care. 2009. https://www.who.int/publications/i/item/9789241594950. 23926623

[r24] WHO. *Complementary Feeding*. Geneva; 2003.

[r25] National Planning Commission. Health Sector Component of National Food and Nutrition Policy: National Strategic Plan of Action for Nutrition 2014–2019. Abuja; 2014. https://faolex.fao.org/docs/pdf/nig158612.pdf.

[r26] National Population Commission [Nigeria] and ICF. 2018 Nigeria Demographic and Health Survey Key Findings. Abuja; 2019. https://dhsprogram.com/pubs/pdf/FR359/FR359.pdf.

[r27] Ibeanu V, Onyechi U, Ani P, Omeh O. Dietary pattern, anthropometric indices and developmental milestone of children aged 6–60 months in peri-urban communities east of Nigeria. Int J Child Health Nutr. 2018;7:22–29.

[r28] Lawan U, Adamu A, Envuladu E, Akparibo R, Abdullahi R. Does maternal education impact infant and child care practices in African setting? The case of Northern Nigeria. Sahel Med J. 2017;20:109–116.

[r29] Mesfin F, Yemane B, Worku A. Prevalence and associated factors of stunting among primary school children in Eastern Ethiopia. Nutr Diet Suppl. 2015;7:61–68.

[r30] Gomwe H, Seekoe E, Goon D, Lyoka P, Marange C. The prevalence of underweight, overweight and obesity among primary school learners in the Eastern Cape Province, South Africa. Pak J Nutr. 2019;18:644–649.

[r31] Bourne P. Childhood health in Jamaica: Changing patterns in health conditions of children 0–14 years. N Am J Med Sci. 2009;1(4):160–168. 22666690 10.4297/najms.2009.4160 PMC3364660

[r32] Hien N, Hoa N. Nutritional status and determinants of malnutrition in children under three years in Nghean, Vietnam. Pak J Nutr. 2009;8(7):958–964.

[r33] Khan T, Khan R, Raza M. Gender analysis of malnutrition: A case study of school-going children in behaviour. Asian Dev Policy Rev. 2015;3(2):29–48.

[r34] Mwangome M, Fegan G, Mbunya R, Prentice A, Berkley J. Reliability and accuracy of anthropometry performed by community health workers among infants under 6 months in rural Kenya. Trop Med Int Health. 2012;17:622–662. doi:10.1111/j.1365-3156.2012.02959.x. 22364555 PMC3963456

[r35] Treffers PE. Teenage pregnancy, a worldwide problem (in Dutch; Flemish). Ned Tijdschr Geneeskd. 2003;147(47):2320–2325. 14669537

[r36] Habitu YA, Yalew A, Bisetegn TA. Prevalence and factors associated with teenage pregnancy, Northeast Ethiopia, 2017: A cross-sectional study. J Pregnancy. 2018; 2018:1714527. doi:10.1155/2018/1714527. 30515326 PMC6236922

